# Managing thrombosis of arteriovenous fistula accompanied by aneurysm and single outflow of elbow perforating vein: a case report

**DOI:** 10.1186/s12882-022-02897-2

**Published:** 2022-07-30

**Authors:** Ziqiang Wang, Yifen Hong, Zefeng Wei, Juan Chen, Li Li, Yongjun Zhu

**Affiliations:** 1 Department of Nephrology, The First Affiliated Hospital of Hainan Medical University, Hainan province, Haikou, 570102 China; 2 Department of Nephrology, The People`S Hospital of Wanning, Hainan province, Wanning, 571500 China

**Keywords:** Arteriovenous fistula, Thrombosis, Single outflow of elbow perforating vein, Aneurysm, Open surgery

## Abstract

**Background:**

We report a case of a patient who suffered thrombosis of a radial artery-cephalic vein fistula accompanied by aneurysm and a single outflow path of the elbow perforating vein. We performed open surgery combined with Fogarty balloon catheter embolectomy, anastomotic reconstruction and forearm median vein transposition.

**Case presentation:**

The patient presented with an arteriovenous fistula (AVF) after haemodialysis 5 years ago. In the process of dialysis, the fistula vein was punctured, resulting in aneurysm, high pressure and difficult haemostasis after needle extraction. AVF occlusion was observed on April 12, 2022. We performed a combined open surgery. First, a Fogarty balloon catheter was used to remove the thrombus, and the anastomosis was then reconstructed to restore AVF fistula patency. Finally, forearm median vein transposition was used to establish dual outflow. Postoperative haemodialysis was possible. There are various methods for removing the thrombus in AVF. Here, we report a case in which we performed open surgery combined with Fogarty balloon catheter embolectomy, anastomotic reconstruction and forearm median vein transposition to ensure fistula patency.

**Conclusion:**

We removed a complete reverse ‘Z’-shaped thrombus of the elbow perforating vein in a haemodialysis fistula. This report provides an effective strategy to manage a high-pressure fistula with single outflow of the elbow perforating vein.

## Background

Autologous arteriovenous fistula (AVF) has been the ideal option for haemodialysis vascular access for decades. During dialysis, fistulas are repeatedly punctured, resulting in aneurysm, and some stenosis or occlusion appears [1]. Stenosis in the venous outflow causes an alternative route of blood flow via the deeper venous pathways by means of perforating veins (PVs) and side branches [2]. Fistula thrombosis also easily occurs after haemodialysis. Thrombosed AVFs can be managed surgically or by less invasive endovascular techniques with or without Fogarty balloon catheters [3]. Percutaneous transluminal angioplasty (PTA) and fistula reconstruction surgery are therapeutic options for vascular access occlusion in haemodialysis patients [4]. The thrombosed fistula with a single outflow of elbow perforating vein makes it difficult to remove the thrombus from the perforating vein due to the reverse ‘Z’ shape. Fistulas with aneurysms are prone to residual thrombosis due to enlargement of the tube diameter, resulting in treatment difficulties. Here, we report a case of a patient who suffered thrombosis of the AVF with an aneurysm and a single outflow path of the elbow perforating vein. The thrombus in the aneurysm and perforating vein were removed successfully, and dual outflow of the fistula was established. The patient avoided central venous intubation.

## Case presentation

**Fig. 1:**
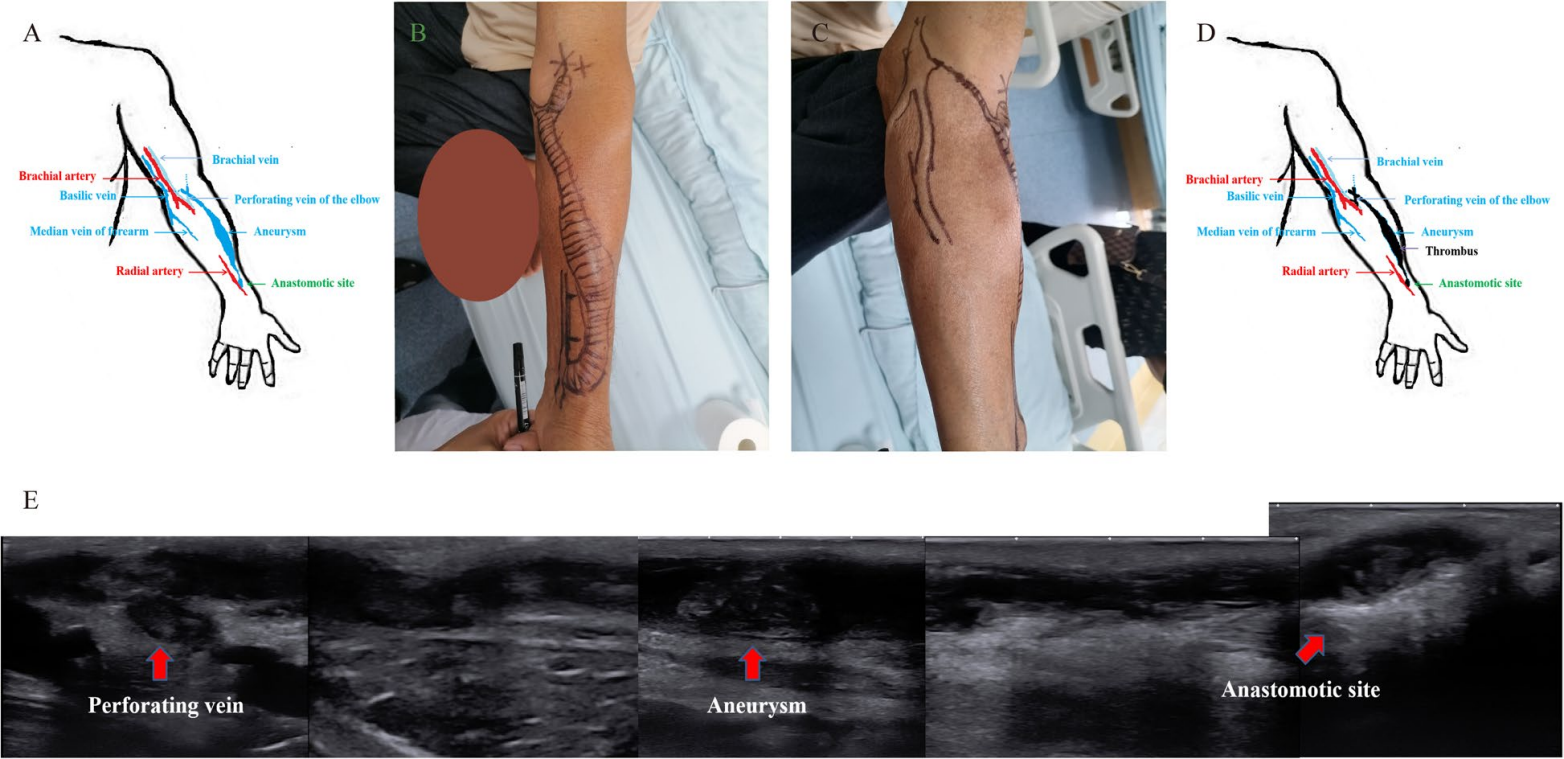
Simplified diagram of the haemodialysis access in the present case (**A**). Photograph of the left upper limb (**B** and **C**). Simplified diagram of the thrombosed arteriovenous fistula (**D**). The thrombus was identified in the vein from the anastomotic site to the elbow using colour ultrasound (**E**)

A 63-year-old man was diagnosed with end-stage renal disease (ESRD) 5 years ago, and an AVF was created at the left wrist for haemodialysis. Aneurysm formation began approximately one year ago and gradually enlarged. Pulsatility was felt in the vein even with palpation. There was also high venous pressure during dialysis and difficulty in haemostasis after puncture, which suggested that the vein was chronically under high pressure. the median cephalic vein and median basilic vein were occluded by physical examination and colour Doppler ultrasound. The fistula had a single outflow path of the elbow perforating vein. Unfortunately, no colour ultrasound images at this time were taken. The state of flow occurred from the perforating vein to the brachial vein (Fig. 1A). The patient did not receive any treatment. Left forearm AVF occlusion was observed on April 12, 2022. A thrombus was observed in the vein from the anastomotic site to the elbow using colour ultrasound. The vein appeared as an aneurysm. Thrombosis also occurred in the perforating vein and brachial vein (Fig. 1B-E).

The cause of thrombosis may have been due to lack of outflow. The patient needed to continue haemodialysis regularly. To restore vascular access in time, we performed an emergency thrombectomy.

**Fig. 2:**
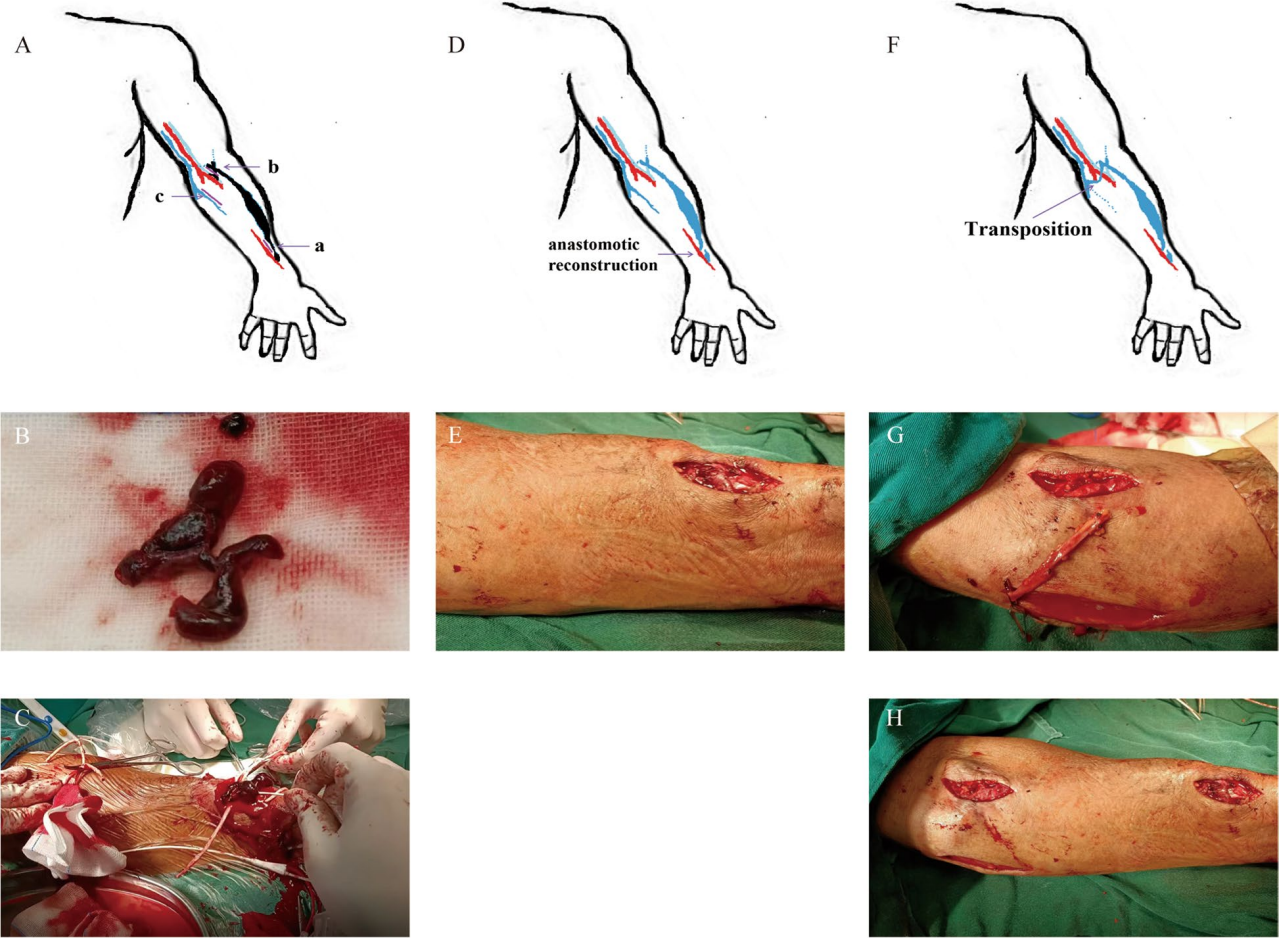
Simplified diagram of the incisions made during the operation (**A**). Photograph of the reverse ‘Z’-shaped thrombus in the elbow perforating vein and the thrombus in the aneurysm (**B** and **C**). Simplified diagram and photograph of anastomotic reconstruction (**D** and **E**). Simplified diagram and photograph of the forearm median vein transposition (**F** and **G**). Photograph at the end of the operation (**H**)

First, we made two longitudinal incisions in the cephalic vein after anastomosis and the median cubital vein. Each incision was approximately 8–10 mm long. We removed the thrombus using haemostatic forceps. The reverse ‘Z’-shaped thrombus of the perforating vein was completely removed (Fig. 2B). Because blood was flowing out, we temporarily cut off the flow of the perforating vein. The thrombus in the aneurysm was removed with a 5.5 F Fogarty balloon catheter (Edwards Lifesciences) guided by colour ultrasound (Fig. 2C). This process was performed several times. We injected a saline solution mixed with heparin (1 mg/ml) into the vein to prevent thrombosis. There was a stenosis segment at approximately 2 cm after the AVF anastomosis, and the diameter of the most stenotic point was only 0.16 cm. Therefore, we performed anastomotic reconstruction to ensure long-term patency but not PTA. The anastomosis was reconstructed at the ‘a’ site, and the radial artery incision was approximately 6 mm (Fig. 2D, E). The blood flow was then resumed. However, pulsatility was felt in the vein at the aneurysm even with palpation, which suggested that the vein was still under high pressure.

**Fig. 3:**
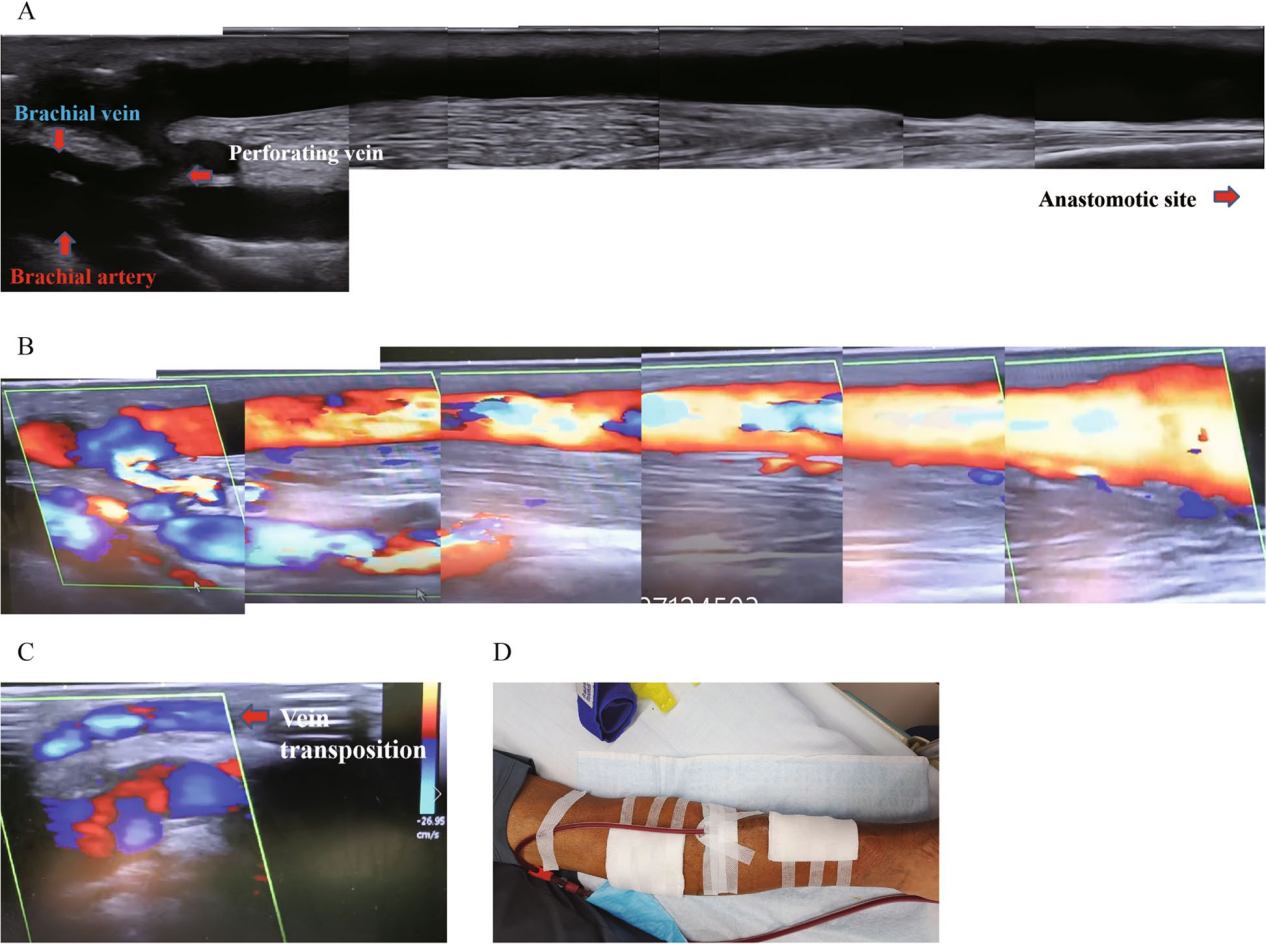
Vascular ultrasound after the operation in 2D and colour mode (**A** and **B**). Colour ultrasound image of the transposed median vein of the forearm (**C**). Photograph of postoperative haemodialysis (**D**)

We attempted an intervention against the occlusion of the median cephalic vein and median basilic vein, but the guide wire did not pass. The diameter of the median vein of the forearm was 3.5 mm as measured by Doppler ultrasound preoperatively. We then transposed the median vein of the forearm to the median cubital vein to establish a dual outflow (Fig. 2F-H). The fistula was re-examined by colour ultrasound, and the blood flow was smooth without obvious residual thrombus (Fig. 3A-C).

Postoperative haemodialysis was possible, and the blood flow was stable (Fig. 3D). The blood flow of the brachial artery at 1 week after surgery was 812 mL/min. When the pump-controlled blood flow rate was 270 ml/min, the venous pressure during dialysis decreased from 110 to 90 mmHg. The time of haemostasis after needle extraction was reduced from 45 to 20 min. Together, these results indicated that the pressure of the fistula was lower than before.

## Discussion and conclusions

Although PTA has been widely used in managing acute thrombosis or stenosis of AVFs, open surgery is inexpensive and improves survival time [5]. In this case, we performed a combined open surgery. We removed the thrombus using haemostatic forceps and a 5.5F Fogarty balloon catheter guided by colour ultrasound. Anastomotic reconstruction was also adopted.

In this case, an embolectomy was insufficient because the outflow needed to be secured. The median cephalic vein and median basilic vein were interrupted and flowed from the perforating vein to the brachial vein. The aneurysm and difficulty in haemostasis after puncture indicated that the vein was under chronic high pressure. The same procedure may be performed using a graft bypass as previously reported [6]. In this case, transposition of the median vein of the forearm was added to establish dual outflow to ensure patency.

Although techniques for embolectomy procedures to establish outflow are common, the reverse ‘Z’-shaped thrombus of the elbow perforating vein in the present case needed to be completely removed. Thus, the present case required simultaneous treatment with embolectomy and transposition of the median vein of the forearm, which are infrequently utilized techniques. Additionally, similar operations performed by haemodialysis physicians are rare.

## Data Availability

All data generated or analysed during this study are included in this published article.

## Abbreviations

ESRD: End stage renal disease
AVF: Arteriovenous fistula
PVs: Perforating veins
PTA: Percutaneous transluminal angioplasty
